# Herpes

**Published:** 1891-03-28

**Authors:** 


### HERPES.

Of true herpes three distinct varieties are now recognised —(*a*) Herpes labialis and facialis; (*b*) herpes preputialis; (*c*) herpes zoster; herpes cruralis and herpes collaris being less common forms which may be included in these varieties. Herpes iris is a distinct affection, and is a variety of erythema, and therefore does not come under our notice at present.

It is an open question as to whether herpes may be rightly regarded as a specific infection, or whether it is a trophic lesion independent of any micro-organism. Kaposi has concluded that a microbe exists in herpes zoster. There certainly are strong points which may be adduced in favour of his views; thus, herpes runs a more or less definite course, lasting about eighteen to twenty days, during which it passes through its various stages in a remarkably uniform manner, and simulating in this respect the phenomena attending the appearance, maturation, and fading away of some of the characteristic lesions of two undoubtedly specific fevers. It is well recognised, too, that herpes zoster rarely attacks a patient twice; this fact, taken in connection with Kaposi's observations on the occurrence of epidemics of the disease, which it seems recur in Vienna every spring and autumn, strongly supports his views. He has also noted that in different epidemics the disease manifests itself in varying severity; in some a mild type prevails, in others the cases are more severe.

But whatever may be said in favour of herpes zoster being an infectious disease, one point will tend to distinguish it from herpes facialis, for this has a great tendency to recur, sometimes recurring every spring. The almost invariably unilateral appearance of herpes facialis and herpes zoster is a fact which cannot be explained unless the lesion is primarily a neurosis.

